# The evaluation of black carrot, green cabbage, grape, and apple juices as substrates for the production of functional water kefir‐like beverages

**DOI:** 10.1002/fsn3.4293

**Published:** 2024-06-24

**Authors:** Bilal Agirman, Ilker Yildiz, Suleyman Polat, Huseyin Erten

**Affiliations:** ^1^ Department of Food Engineering, Faculty of Engineering Cukurova University Adana Türkiye

**Keywords:** aroma, black carrot, fermentation, functional beverage, green cabbage, water kefir

## Abstract

Water kefir (WK) is a nondairy probiotic beverage produced using water kefir grains that are highly adaptable to diverse food substrates. Fruit and vegetables have been used more in beverage production in recent years due to their plentiful nutritional qualities. In this context, the aim of this study is to develop fruit–vegetable juice‐based beverages fermented with WK grains in order to produce novel, non‐dairy, probiotic water kefir‐like beverages (W‐KLBs) with improved sensory and nutritional properties. In this context, black carrot (BC), apple (A), grape (G), and green cabbage (GC) juices are fermented with commercial WK grains. Results showed that BC‐KLB possessed the highest antioxidant activity (75.50%), total phenolic (1248.60 mg GA/L), and total monomeric anthocyanin (391.31 mg/L as cyaniding‐3‐glucoside equivalent) content. Also, the sensory evaluation demonstrated that BC‐KLB was the most favorable sample, while GC‐KLB received negative feedback. These findings strongly support the suitability of BC juice to develop W‐KLB with high added value and functional properties.

## Graphical abstract

The experiments were conducted using black carrot, green cabbage, apple, and grape juices as substrates for fermentation, employing water kefir grains as the fermenting agent. Water kefir‐like beverage (W‐KLB) derived from black carrot juice exhibited the highest functional characteristics with favored sensorial attributes. Due to the abundance of sulfur compounds in its aroma, green cabbage is not suitable for the production of W‐KLB. Beverages produced with grape and apple juice showed a similar aroma and sensory profile.

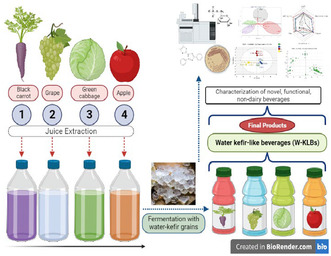

## 1 INTRODUCTION

Increasing consumer interest in health and well‐being has led to a shift in recent years toward functional foods and beverages (Ganatsios et al., 2021). As a direct result of this, there is a lot of interest in creating new kinds of functional foods (Ozcelik et al., 2021; Parades et al., 2022). This food category encompasses all healthful probiotic foods, with traditional fermented beverages such as milk kefir and water kefir (WK) gaining particular popularity (Pendon et al., 2022). Due to the dairy allergies of some individuals, nondairy foods have been the subject of significant investigation in recent years (Cosme et al., 2022; Mishra et al., 2021; Parades et al., 2022; Sherman et al., 2024). Also, since vegetarianism is a growing trend and there are more vegans and vegetarians, veganism has become an important dietary choice (Yerlikaya et al., 2022). As an alternative to dairy products, WK is a crucial nondairy fermented beverage typically produced by inoculating WK grains (WKG) into a sugar (sucrose)‐rich solution added with dried fruits like figs and raisins (sometimes with a slice of lemon) (Patel et al., 2022). WKG range in size from a few millimeters to a few centimeters and are typically smooth, translucent, gray‐white in color, waxy, tough in consistency, and can be described as resembling “rock salt” (Guzel‐Seydim et al., 2021). Unlike milk kefir, the polysaccharide matrix of WKG is primarily constituted of dextran (about 95%–97%), with the grains containing a diverse consortium of lactic acid bacteria (LAB), acetic acid bacteria (AAB), and yeasts embedded in the dextran matrix (Calatayud et al., 2021; Tzavaras et al., 2022).

WK is described as a sparkling, carbonated, acidic, low‐alcohol fermented beverage with a sweet taste. Current research reveals that many of the LAB, bifidobacteria, and yeasts that comprise the microbiota of WKG have a symbiotic relationship and exhibit probiotic characteristics (Cufaoglu & Erdinc, 2023; Laureys & De Vuyst, 2014). Therefore, the presence of probiotic microorganisms is linked to the health benefits of WK when sufficiently consumed. The functional properties of WK are noted in many studies. In this context, the health benefits of WK, such as its antimicrobial, anti‐inflammatory, anticarcinogenic, antidiabetic, antimicrobial, antioxidant, hepatoprotective, and wound‐healing effects, have been demonstrated in various studies (Cufaoglu & Erdinc, 2023; Guzel‐Seydim et al., 2021). Furthermore, WK may benefit people with lactose intolerance by improving the immunological and gastrointestinal systems, reducing cholesterol, as well as having high antioxidant capacity and prebiotic properties (Cai et al., 2020; Tzavaras et al., 2022). In the food market, there is an increasing demand for foodstuffs with health benefits from customers. In recent years, nondairy substrates such as fruit and vegetable juices, molasses, desserts, and cereal‐based products have been regarded as promising food matrices for the development of novel probiotics with antioxidant and antiaging effects (Ozcelik et al., 2021; Tireki, 2022). Nowadays, there is a considerable interest in developing kefir‐like beverages (KLBs) with improved functional characteristics by fermenting generally fruit and sometimes vegetable juices with WKG (de Souza et al., 2024). Official authorities highly urge the consumption of fruits and vegetables due to their functional features, such as lowering the risk of numerous diseases and antiaging. Fruit and vegetable juices can aid in fermentation and serve as probiotic carriers because they include high quantities of sugars, dietary fiber, and other components with considerable nutritional value, such as antioxidant polyphenolics, which make them readily acceptable to consumers (Cai et al., 2020). Fermentation is one method of processing fruits and vegetables. It is anticipated that fermentation of fruit and vegetable juices will result in the development of healthy new product options for customers (Ozcelik et al., 2021). Instead of sucrose‐containing water, many different substrates such as soy whey (Azi et al., 2020; Tu et al., 2019), green coconut water (Dwiloka et al., 2020), palms sap (Zongo et al., 2020), red pitaya and apple pulp (Bueno et al., 2021), olive juice (Darvishzadeh et al., 2021), carrot (Rejdlová et al., 2023), fennel, melon, onion, tomato, strawberry (Corona et al., 2016), kiwifruit, prickly pear, pomegranate, grape, quince, apple (Randazzo et al., 2016), pumpkin (Koh et al., 2018), honey, and grape molasses (Çevik et al., 2019) have been used in fermentation to produce water KLBs. There has been no research into fermenting black carrot juice (BCJ) with water kefir grains, and as far as we know, only one investigation has been conducted on fermenting BCJ with milk kefir culture (Kabakcı et al., 2022).

In view of the many health benefits of both kefir and vegetables and fruits, the goal of this study is to assess the use of juices extracted from fresh apples, grapes, black carrots, and green cabbage as a substrate for the manufacture of new nondairy functional KLBs fermented via probiotic strains originating from WKG. To our knowledge, it is the first time that the juices of black carrot and green cabbage were fermented by WKG to produce KLBs. The proximate, physicochemical, microbiological, and sensorial characteristics were determined. The KLBs were also compared in terms of their aroma profiles.

## 2 MATERIALS AND METHODS

### 2.1 Materials

The fresh WKG involved in this study was provided by Danem Inc. (https://www.kefirdanem.com/) in Isparta, Türkiye. In this study, three fruits and one vegetable were used. Apples (*Malus domestica* Borkh, cv Starking Delicious), grapes (*Vitis vinifera* L., cv Sultana Seedless), black carrots (*Daucus carota* ssp. *sativus* var. *atrorubens* Alef., cv Türkiye), and green cabbage (*Brassica oleracea*, cv capitata) were purchased from a local market in Adana, Türkiye.

### 2.2 Production of KLBs

Black carrots, apples, grapes, and green cabbage were first sorted and then washed. The juices were obtained using a centrifugal extractor (Philips HR1856/70, Netherlands) and pasteurized at 75°C for 5 min. WKG were reactivated by back‐slopping using sterilized water, including 10% of sucrose, and two whole figs in small slices. Back‐sloping was performed three times at 25°C for 48 h. The grains were subsequently filtered via a sterile sieve and rinsed with sterile distilled water. To start the fermentation, 3 g of the activated fresh WKG were weighed into 3 L of corresponding juices (1:1000 w/v). The fermentation process was statically performed at 25°C for 48 h. KLB productions were conducted in 3 L sterilized (at 121°C for 15 min) glass bottles in triplicate.

### 2.3 General analysis

The pH, total titratable acidity (TTA), and density of the juices were measured before, during, and after fermentation. The pH of the samples was detected directly using a digital pH meter (Seven Compact, pH/Ion S220). The TTA level of the samples was found by titrating the samples with 0.1 N NaOH in the presence of phenolphthalein indicator to pH 8.1 and expressing the results as g/L lactic acid. The density value of the juices was determined by a portable density meter (Mettler Toledo, Densito 30PX) at 20°C.

Total soluble solids (TSS) or°Brix were determined by measuring the refractive index of fermented KLBs with a hand refractometer (KEM Kyoto, RA‐130, Tokyo, Japan). Results were expressed as percentages (sucrose g/100 g juices).

### 2.4 Ethanol content

The alcohol concentration in W‐KLBs was determined using near‐infrared spectroscopy (Anton Paar, DMA™ 4500 M) through the oscillating U‐tube method. Following the CO_2_ removal and filtering steps, a U‐shaped borosilicate glass tube was automatically filled with 50 mL of homogenized sample (at 20°C) using an automatic sample filling unit. Various alcohol tables (OIML, AOAC, IUPAC, etc.) that are programmed into the instrument allow for the direct display of alcohol concentration by volume (% v/v). The principle behind this officially recognized method is to measure density first, then convert it to alcohol concentration using official alcohol tables.

### 2.5 Color analysis

Color of KLBs was directly measured after fermentation according to CIElab chromaticity coordinates (L*, a*, b*) by ColorQuest XE (HunterLab, Virginia, USA) spectrophotometer. Measurement parameters were standardized to daylight illumination and 10‐degree standard observer (D65/10°) (Agirman et al., 2021). Analysis was performed in TTRAN mode, and a black card was placed flat against the transmission port for standardization. In addition, a white ceramic tile was inserted and left at the reflectance port to maintain a consistent white background during measurements. The average values of three basic chromatic coordinates were calculated: L* brightness (dark, medium, bright, L* = 0 for black, and L* = 100 for white), a* red/green (+a* indicates red and −a* indicates green), and b* yellow/blue (+b* indicates yellow and −b* indicates blue), which were then used to calculate chroma (*C*_ab_) and hue angle (h°) values. The C* value represents the color's strength (saturation; low, medium, or high) or purity, whereas the h° shows the product's main color perceived (red, yellow, green, and blue, or a combination of adjacent pairs of these colors) (CIE, 2011). *C*_ab_ and h° parameters were indirectly calculated as follow: *C*_ab_ = (a*^2^ + b*^2^)^1/2^, h° = arctan(b*/a*).

### 2.6 Microbiological analyses

Unpasteurized fruit–vegetable juices (U‐FVJs), WKG, and fermented KLBs were investigated by plate count for the enumeration of several microbial groups. For the microbial analyses of WKG, 2 g of grain was placed into a sterile stomacher bag including 18 mL of sterile peptone water. The grains were then crushed in a Bag Mixer (Intersience, Model 400P, France) for 3 min and kept for 1 h to allow for the transmission of microorganisms to the dilution solution. Microbiological investigations were performed on each day (0, 1, and 2) of fermentation to estimate the change in microbial groups during the production of KLBs from various substrates. For zero‐day analysis, specimens were taken 2 h after the start of fermentation. Following the homogenization of the fermentation vessel, 1 mL of each sample was taken and serially diluted in a test tube containing 9 mL of physiological saline solution. Diluted samples were spread onto agar plates containing corresponding selective media for each microbial group. Cell suspensions were plated and incubated as follows (Cirak et al., 2022; Randazzo et al., 2016): total mesophilic count (TMC) spread plated on plate count agar (PCA) incubated aerobically at 30°C for 72 h; coliform bacteria (CB) pour plated on violet red bile agar (VRBA) incubated aerobically at 37°C for 48 h; rod LAB pour plated on de Man–Rogosa–Sharpe (MRS) agar supplemented with cycloheximide (100 μg/mL, Sigma‐Aldrich) incubated anaerobically at 30°C for 48 h; coccus‐LAB pour‐plated M17 agar supplemented with cycloheximide (100 μg/mL, Sigma‐Aldrich) incubated anaerobically at 30°C for 48 h; total yeasts (TY) spread plated on potato dextrose agar (PDA) supplemented with oxytetracycline (100 μg/mL, Sigma‐Aldrich) incubated aerobically at 25°C for 72 h, and non‐*Saccharomyces* yeasts spread plated on L‐Lysine agar supplemented with oxytetracycline (100 μg/mL, Sigma‐Aldrich) incubated aerobically at 25°C for 72 h. Colony counts were expressed as log cfu/mL at the end of the incubation period.

### 2.7 Determination of total phenolic content (TFC) and total monomeric anthocyanin (TMA)

The TFC of the KLBs was determined according to Folin–Ciocalteu method previously described by Agcam (2022). The absorbance of centrifuged and clarified samples was detected at 765 nm using a UV/VIS spectrophotometer (Perkin Elmer Lambda 25, USA). The TPC of the beverages was quantified using a calibration curve derived from different concentrations of gallic acid solution. The results were presented as milligrams of gallic acid equivalents per liter of KLBs (mg GAE/L).

TMA content was determined using the pH differential method suggested by Lee et al. (2005). This technique is based on the reversible structural transformation of anthocyanins as a function of pH changes (colored oxonium form at pH 1.0 and colorless hemiketal form at pH 4.5). Therefore, the difference in absorbance at the pigment's maximum wavelength (λ_vis‐max_ 520 nm) is proportional to its concentration. Readings were recorded at 520 and 700 nm on a zeroed UV/VIS spectrophotometer (Perkin Elmer Lambda 25, USA) against distilled water. The following formula was used to quantify the TMA content of the samples:

(1) $$A={\left(A_{\lambda_{520\mathrm{nm}}}-A_{\lambda_{700\mathrm{nm}}}\right)}_{\mathrm{pH}=1}-{\left(A_{\lambda_{520\mathrm{nm}}}-A_{\lambda_{700\mathrm{nm}}}\right)}_{\mathrm{pH}=4.5}$$

(2) $$\mathrm{TMA}\;\left(\mathrm{mg}/L\right)=\frac{A.\mathrm{MW}.\mathrm{DF}}{Ɛ.l}\times 1000$$

where *A* is absorbance value, MW is the molecular weight of cyanidin‐3‐glucoside (449.2 g/mol) which is the most common anthocyanin pigment found in nature, DF is dilution factor, Ɛ is the molar extinction coefficient (29,600 L/cm.mol), and l is the path length (1 cm) of the cuvette. TMA results were expressed as mg cyanidin‐3‐glucoside equivalents per liter of KLBs and all measurements were replicated three times.

### 2.8 Antioxidant activity (AA)

The antioxidant activity of the samples was determined by 2,2‐diphenyl‐1‐picrylhydrazyl (DPPH) radical scavenging activity as previously described by Klimczak et al. (2007). After incubating at 30°C for 60 min in the dark to establish equilibrium with the reaction, the absorbance was measured at 517 nm. AA was calculated as a percentage (%) of the decline in absorbance using the subsequent formula:

(3) $$\mathrm{AA}\;\left(\%\right)=\left(\frac{A_{\text{control}}-A_{\text{sample}}\;}{A_{\text{control}}}\;\right)\times 100$$

### 2.9 Identification and quantification of volatile compounds (HS‐SPME‐GC–MS)

Aroma components from KLB samples have been obtained using a combination of four analytical techniques: headspace, solid‐phase microextraction, gas chromatography, and mass spectrometry (HS‐SPME‐GC‐MS), as detailed in Sevindik's (2020) paper. The protocol was as follows: 3 mL of fruit beverage samples were placed in a 20 mL vial, and 5 μL of the internal standard (IS: 4‐nonanol) was added. The glass vial was then screwed shut and sealed with a PTFE‐silicon septum. Afterward, the vial was transferred into the heating panel by an automatic sampler and preheated for 15 min at 60°C. Then, a 2 cm DVB/CAR/PDMS (divinylbenzene, carboxen, and polydimethylsiloxane) fiber (50/30 μm) (Supelco, Bellefonte, PA, USA) was automatically inserted into the head space of the vial, and accumulated volatiles were retrieved for 45 min at 60°C. Desorption of absorbed volatile substances from the fiber was accomplished by inserting the fiber into the instrument's injection port for 2 min at 250°C. For the aroma analysis of the samples, an Agilent gas chromatograph connected to an Agilent 7000D triple‐quadrupole MSD and interfaced with a flame ionization detector (FID) was employed. The volatile compounds were separated utilizing a DB‐Wax capillary column (30 m × 0.25 mm, 0.25 μm film thickness). A 3 μL of extract was injected in splitless pulse mode at 40 psi for half a minute. For the injector and FID detectors, temperatures of 270 and 280°C, respectively, were utilized. The flow rate of the carrier gas (helium) was 1.5 mL/min. The program parameters for the DB‐Wax column oven were set to 50–250°C at a rate of 4°C/min with a 10‐min hold. These oven parameters were used for the MSD as well. MS parameters included a scan rate of 2.0 scan/s, 70 eV ionization energy, mass range m/z of 35–400 amu, 250°C interface temperature, and 250°C source temperature.

The volatiles in the samples were identified based on their retention indices (RIs), internal standard compound, and mass spectra on the DB‐Wax column using a commercial database of spectra (NIST 11, Wiley 9.0, Flavor 2 L). These identifications were then verified using the external standard. To construct standard calibration curves for quantification, the peak ratios of the target compound to the internal standard were plotted against the concentration. Volatile substances with no reference standards were approximately quantified using standards with identical functional groups and/or a similar number of carbon atoms (Sevindik et al., 2022).

### 2.10 Sensory evaluation

The final beverages were subjected to sensory analysis by 13 untrained judges (six females and seven males, aged 25–48). The study was carried out in compliance with the Declaration of Helsinki criteria and received approval by the Ethics Committee of Çukurova University's Food Engineering Department (protocol codes 2020–17 and January 14, 2020). Sensory analysis was carried out in a sensory analysis laboratory affiliated with the Department of Food Engineering at Cukurova University, Turkey. The design of the sensory analysis room was standardized in accordance with ISO 8589:2007 (sensory analysis, general guidance for the design of test rooms). Each panelist was separated from the others by an individual sensory evaluation booth, and the sensory assessment was conducted under neutral white light (4000 K). Each beverage was refrigerated and served (at 8 ± 1°C) at random in clear glass cups containing 20 mL of samples. Each panelist received five samples (four W‐KLBs and one control sample) per session and rated them on six characteristics using a 9‐point hedonic scale in a sensory room, with 9 being the most pleasant and 1 being the most unpleasant. The attributes were as follows: acidity, fragrance and aroma, alcohol perception, viscosity, taste, and overall acceptance. A water kefir, produced with the same microbial mixture, was used as control to compare the fruit kefir beverages. In each session, five samples were examined and each product was evaluated in triplicate (Muir et al., 1999). A preference test was also conducted as a second sensory test. In this test, panelists were asked to rank four W‐KLBs and one control sample in descending order of preference or liking (Lawless & Heymann, 2010). This test was applied to the same group of panelists, and they were asked to rank the samples from most preferred to least preferred using the following numbers (1 = most preferred, 5 = least preferred) with a preference test (ranking) form. Cukurova University examined and approved the sensory analysis study, and informed consent was obtained from each assessor prior to their participation in the study.

### 2.11 Statistical analysis

The data were analyzed using one‐way analysis of variance (ANOVA), and the differences in mean values were assessed using post doc Tukey's method (*p* < .05). Statistical data were processed by SPSS Statistics 23.0 Software (IBM Corp., Armonk, NY, USA). Moreover, aroma compounds were normalized and subjected to principal component analysis (PCA) to identify the primary volatiles responsible for KLB distinction. Pearson (n‐1) was selected as PCA type, and analysis was performed using the XLSTAT version 2019.2.2. Partial least squares‐discriminant analysis (PLS‐DA) was performed by SIMCA, version 14.1 (Umetrics, Umea, Sweden).

## 3 RESULTS AND DISCUSSION

### 3.1 pH, total titratable acidity, and density

Fermentation was monitored daily by following pH, TTA, and density parameters. The results of the chemical determinations are given in Table 1. It is critical to note that various fruits and vegetables have distinct chemical and nutritional compositions, such as varying levels of sugar and pH. In this study, black carrot and green cabbage juice were characterized by high initial pH values (above 6.0) and low TTA content (below 1.50 g/L), whereas grape and apple juice showed quite lower initial pH values (below 4.0) and reasonably higher TTA levels (above 3.00 g/L). Generally, the pH and sugar content of fruits affect the activity of microorganisms (Mohideen et al., 2015). As a result, the high initial pH of black carrot and green cabbage juices can explain the high microbial counts (Table 2) observed in these juices prior to fermentation. Fermentation of black carrot, grape, apple, and green cabbage juices with WKG significantly changed the KLB's pH and TTA values (*p* < .05). At the end of the fermentation, there were only slight variations in the initial pH values in the grape (0.2 unit) and apple juice (0.3 unit) experiments, whereas there were considerable decreases in the black carrot (2.29 unit) and green cabbage (2.42 unit) fermentations. On the other hand, TTA levels increased significantly during the first and second days of fermentation in all trials (*p* < .05). The lowest and highest TTA levels were detected in apple juice‐based KLB and grape juice‐based KLB as 8.16 and 13.08 (g/L lactic acid), respectively. According to Randazzo et al. (2016), the TTA level of water KLBs from apple and grape juice was 2.35 g/L citric acid (equivalent to 3.31 g/L lactic acid) and 2.91 g/L citric acid (equivalent to 4.09 g/L lactic acid), respectively. There may be two reasons why they obtained lower acidity than we did: first, they used freeze‐dried microbial culture instead of fresh kefir grains, and second, there are still plenty of fermentable sugars in the medium because the brix degree (sugar content) of the final product is quite high. The pH levels in the apple (4.04)‐ and grape (3.81)‐based KLBs in the same study were similar to the current study. To the best of our knowledge, there are no comparable studies on the production of a water kefir‐like beverage (W‐KLB) from green cabbage and black carrot juice. However, it has been reported that the TTA level in Şalgam, a traditional Turkish lactic acid‐fermented beverage made from black carrots with characteristics similar to water KLBs, varies between 7.40 and 8.71 g/L as lactic acid (Agirman & Erten, 2018).

**TABLE 1:** Physicochemical composition of KLB during fermentation and in the final product.

	Fermentation days	G‐KLB	BC‐KLB	A‐KLB	GC‐KLB
pH	Day 0	3.89 ± 0.00^d^	6.32 ± 0.02^a^	3.96 ± 0.00^c^	6.01 ± 0.01^b^
	First day	3.71 ± 0.01^c^	4.07 ± 0.00^a^	3.84 ± 0.01^b^	3.76 ± 0.01^c^
	Second day	3.87 ± 0.00^b^	4.03 ± 0.00^a^	3.99 ± 0.01^ab^	3.59 ± 0.05^c^
TTA (g/L)	Day 0	4.37 ± 0.03^a^	1.49 ± 0.03^c^	3.19 ± 0.06^b^	1.01 ± 0.13^d^
	First day	7.40 ± 0.52^ab^	8.39 ± 0.70^a^	4.67 ± 0.19^b^	7.45 ± 1.24^ab^
	Second day	13.08 ± 0.97^a^	8.42 ± 0.90^b^	8.16 ± 1.39^b^	9.23 ± 0.60^ab^
Density (g/cm^3^)	Day 0	1.091 ± 0.00^a^	1.038 ± 0.00^c^	1.051 ± 0.00^b^	1.017 ± 0.00^d^
	First day	1.050 ± 0.00^a^	1.009 ± 0.00^d^	1.022 ± 0.00^b^	1.015 ± 0.00^c^
	Second day	1.000 ± 0.00^c^	1.009 ± 0.00^b^	1.000 ± 0.00^c^	1.015 ± 0.00^a^
TFC (mg/L)	E.P.	190.42 ± 6.94^bc^	1248.60 ± 95.28^a^	290.85 ± 6.08^b^	107.22 ± 2.02^c^
TMA (mg/L)	E.P.	0.00 ± 0.00^b^	391.31 ± 41.95^a^	0.00 ± 0.00^b^	0.00 ± 0.00^b^
AA (%)	E.P.	26.68 ± 1.01^c^	75.50 ± 0.95^a^	44.44 ± 4.53^b^	10.66 ± 3.52^d^
L*	E.P.	82.32 ± 0.57^ab^	1.03 ± 0.12^c^	61.74 ± 11.22^b^	90.06 ± 0.28^a^
a*	E.P.	2.33 ± 0.05^ab^	7.03 ± 0.94^a^	7.96 ± 3.68^a^	−1.39 ± 1.13^b^
b*	E.P.	18.55 ± 0.90^b^	1.61 ± 0.19^c^	47.91 ± 2.17^a^	3.88 ± 3.75^c^
*C*_ab_	E.P.	18.70 ± 0.90^b^	7.21 ± 0.95^c^	48.63 ± 2.74^a^	4.54 ± 2.85^c^
h°	E.P.	82.82 ± 0.18^ab^	12.89 ± 0.19^b^	80.55 ± 3.87^ab^	109.80 ± 39.39^a^
Brix (%)	E.P.	6.85 ± 1.48^a^	6.95 ± 0.07^a^	4.90 ± 0.14^a^	5.00 ± 0.28^a^
Ethanol (% v/v)	E.P.	4.80 ± 0.01^a^	0.90 ± 0.00^c^	1.80 ± 0.01^b^	0.14 ± 0.00^d^

*Note*: Results are the means of three measurements for each replicate. Different letters within the same row represent statistically significant differences (*p* < .05). Day 0 analyses were performed on juices freshly extracted just before the trials were set up.

Abbreviations: AA, antioxidant activity; E.P., end product; G‐KLB, BC‐KLB, A‐KLB, and GC‐KLB, grape juice, black carrot juice, apple juice, and green cabbage juice‐based kefir‐like beverages, respectively; SG, specific gravity; TFC, total phenolic content (as gallic acid equivalent mg/L); TMA, total monomeric anthocyanin (as cyanidin‐3‐glucoside equivalent mg/L); TTA, total titratable acidity (as lactic acid g/L).

**TABLE 2:** Microbial loads of water kefir‐like beverages.

Microorganisms (log cfu/mL)							
Sample		TMC	TY	Non‐*Sacc*.	Rod‐LAB	Coccus‐LAB	CB
Unpasteurized FVJs							
Grape		4.95 ± 0.28^c^	3.69 ± 0.40^d^	3.47 ± 0.11^d^	2.00 ± 0.07^d^	3.95 ± 0.22^d^	1.47 ± 0.04^d^
Black carrot		8.02 ± 0.17^b^	7.99 ± 0.08^b^	7.83 ± 0.16^b^	7.81 ± 0.11^b^	7.88 ± 0.35^b^	7.20 ± 0.26^a^
Apple		4.90 ± 0.20^d^	4.11 ± 0.10^c^	4.34 ± 0.38^c^	4.02 ± 0.27^c^	4.25 ± 0.11^c^	2.90 ± 0.20^c^
Green cabbage		8.67 ± 0.69^a^	8.43 ± 0.20^a^	8.90 ± 0.55^a^	8.05 ± 0.38^a^	7.96 ± 0.25^a^	6.04 ± 0.21^b^
Inoculant							
WKG		7.21 ± 0.04	5.56 ± 0.17	4.41 ± 0.04	7.38 ± 0.14	6.21 ± 0.07	<1
Fermented KLBs	Fermentation days						
G‐KLB	Day 0	5.37 ± 0.19^bB^	4.53 ± 0.14^aC^	3.37 ± 0.45^aC^	5.58 ± 0.16^bB^	4.52 ± 0.19^cC^	<1
	Day 1	7.43 ± 0.11^aA^	6.38 ± 0.11^aB^	5.97 ± 0.12^aB^	7.22 ± 0.03^aA^	6.13 ± 0.29^bB^	<1
	Day 2	7.77 ± 0.14^aA^	7.79 ± 0.21^aA^	7.43 ± 0.53^aA^	7.67 ± 0.27^bA^	7.44 ± 0.18^bA^	<1
BC‐KLB	Day 0	5.58 ± 0.11^abC^	4.48 ± 0.05^aB^	3.36 ± 0.04^aB^	6.38 ± 0.09^aB^	4.47 ± 0.11^cC^	<1
	Day 1	7.66 ± 0.02^aA^	6.30 ± 0.06^aA^	5.97 ± 0.23^aA^	7.44 ± 0.31^aA^	6.86 ± 0.34^abB^	<1
	Day 2	6.53 ± 0.08^bB^	6.79 ± 0.13^bA^	6.25 ± 0.03^bA^	8.04 ± 0.26^aA^	8.03 ± 0.18^aA^	<1
A‐KLB	Day 0	5.99 ± 0.41^aB^	3.92 ± 0.34^bC^	3.33 ± 0.13^aC^	6.03 ± 0.36^aB^	5.15 ± 0.22^aB^	<1
	Day 1	7.63 ± 0.49^aA^	6.48 ± 0.04^aB^	5.59 ± 0.11^aB^	6.34 ± 0.19^bB^	7.61 ± 0.08^aA^	<1
	Day 2	7.70 ± 0.11^aA^	7.79 ± 0.15^aA^	7.28 ± 0.11^aA^	7.62 ± 0.21^bA^	7.23 ± 0.22^bA^	<1
GC‐KLB	Day 0	5.52 ± 0.73^abC^	3.77 ± 0.15^bB^	3.38 ± 0.18^aB^	5.75 ± 0.21^bA^	4.90 ± 0.12^bB^	<1
	Day 1	7.01 ± 0.22^bA^	4.38 ± 0.55^bA^	3.53 ± 0.33^bB^	5.79 ± 0.11^cA^	5.48 ± 0.12^cA^	<1
	Day 2	6.06 ± 0.14^cB^	4.37 ± 0.52^cA^	4.03 ± 0.27^cA^	5.26 ± 0.16^cB^	5.22 ± 0.19^cA^	<1

*Note*: For unpasteurized FVJs, values with different superscripts (^a‐d^) in the same column indicate statistically significant differences (*p* < .05) among four different juices (between treatments). For fermented KLBs; ^A‐C^: Uppercase superscripts indicate significant differences between the different fermentation days of the same treatment (*p* < .05), ^a‐c^: Lowercase superscripts indicate significant differences among the same fermentation days of the different treatments (*p* < .05).

Abbreviations: A‐KLB, apple‐based KLB; BC‐KLB, black carrot‐based KLB; CB, coliform bacteria count; coccus‐LAB, number of coccus‐shaped lactic acid bacteria; FVJs, fruit–vegetable juices; GC‐KLB, green cabbage‐based KLB; G‐KLB, grape‐based KLB; KLB, kefir‐like beverage; Non‐*Sacc*., Number of non‐*Saccharomyces* yeasts; rod‐LAB, number of rod‐shaped lactic acid bacteria; TMC, total mesophilic count; TY, total yeast count; WKG, water kefir grain.

Density is a variable with a high interrelation between the density of a juice and its soluble solid content (SSC, mainly sugars). As the SSC increases, density also increases (Rydzak et al., 2020). Grape juice had the highest density value among the freshly extracted juices, followed by apple, black carrot, and green cabbage juices. Fermentation proved to be successful in grape and apple‐based experiments since the entire amount of sugars had been metabolized by microorganisms present in WKG, and the density value reached 1.000 g/cm^3^ at the end of both trials. However, no change in density value was observed between the first and second day of fermentation with black carrot and green cabbage. This result indicates that there is still sugar in the black carrot and green cabbage fermentation medium, but the WKG microbiota is unable to ferment these sugars. Similar density values in fresh juices have been reported as: 1.045 to 1.078 g/cm^3^ for apples (Rydzak et al., 2020), 1.062 to 1.087 g/cm^3^ for grapes (Derradji‐Benmeziane et al., 2014), and 1.032 g/cm^3^ for carrots (Riganakos et al., 2017). There was no comparative study that reported the density of green cabbage juice.

### 3.2 °Brix and ethanol content of water KLBs

The°brix and ethanol values of KLBs are given in Table 1.°Brix is a unit of measurement for the amount of soluble solids in a solution. Despite the fact that sugars, pectins, organic acids, and amino acids are the most common soluble solids in fruit and vegetable juices and all contribute to°brix values, brix readings primarily represent estimations of sugar concentration in fruits and vegetables (Kleinhenz & Bumgarner, 2013). Based on the density values, it can be concluded that the amount of sugar decreased significantly between the beginning and end of fermentation. This is because fermentable carbohydrates are transformed into ethyl alcohol and carbon dioxide during fermentation. The°brix values of water KLBs varied between 4.90% and 6.95% (*p* < .05), whereas the ethanol concentration ranged from 0.14% to 4.80% (*p* < .05). It is remarkable that there is significant variation between the ethanol levels of the KLBs, with the ethanol level in beverages produced using grape and apple juice being significantly higher than in experiments where black carrot and cabbage juices were used as substrate. Since the initial sugar content of green cabbage (1.017 g/cm^3^) and black carrot (1.038 g/cm^3^) juices was low and remained constant after the first 24 h of fermentation, it was assumed that no fermentable sugar remained in the environment after 24 h and hence the ethanol level remained low. However, despite the high starting sugar concentration of grape (1.091 g/cm^3^) and apple (1.051 g/cm^3^) juices, sugar was totally consumed after 48 h (1.000 g/cm^3^) for both fermentation. For these reasons, the alcohol content of G‐KLB and A‐KLB was found to be higher than that of BC‐KLB and GC‐KLB. Notably, ranking the ethanol ratios of the beverages from high to low (G‐KLB, A‐KLB, BC‐KLB, and GC‐KLB, respectively) gives the same order as their initial sugar ratios. GC‐KLB recorded the lowest concentration of ethanol (0.14%) and that is a direct consequence of the limited growth of yeasts, which are primarily responsible for the production of alcohol (Corona et al., 2016). Various sulfur compounds that are naturally present in green cabbage, in particular methyl methanethiosulfinate and allyl isothiocyanate, inhibit the proliferation of yeasts to a significant degree (Kyung & Fleming, 1997). It is hypothesized that this is another factor that reduces alcohol production by preventing yeast growth in water KLB made with green cabbage juice. Results showing low yeast counts in the GC‐KLB in the microbiology section supported this inference.

The presence of ethanol is critical for water kefir products because it imparts the characteristic mild alcoholic flavor and contributes to the unique organoleptic properties of the beverage, along with the lactic acid, acetic acid, and carbon dioxide that are produced during fermentation (Bueno et al., 2021; Guzel‐Seydim et al., 2021). In another study, the alcohol content of the KLB made from grape juice was determined to be high (4.44%), while it was 2.67% in KLB obtained from apple juice (Randazzo et al., 2016). Although there are no studies involving green cabbage and black carrots, the alcohol content of a beverage made with orange carrots was found to be 3.0% (Corona et al., 2016). The brix value of apple (8.70%)‐ and grape (8.47%)‐based KLBs at the end of fermentation was higher in the same studies than it was in this study, while it was lower in carrot (3.38%)‐based KLB.

### 3.3 Results of TFC, TMA, and AA

The TFC of the KLBs produced varied significantly, ranging from 107.22 mg GA/L to 1248.60 mg GA/L (*p* < .05) (Table 1). The same was determined for antioxidant activity values, and the AA capacities of the water KLBs varied from 10.66% to 75.50% (*p* < .05). There was a positive correlation between the total phenolic content and antioxidant activity, as AA increased with increasing TFC in KLBs. As a result, when the TPC and AA capacities of the samples were ranked from high to low, the same order was obtained as BC‐KLB, A‐KLB, G‐KLB, and GC‐KLB, respectively. In accordance with our findings, numerous researchers (Corona et al., 2016; Dani et al., 2007; Ozcelik et al., 2021) have found a substantial relation between TFC and AA in fruit and vegetable juices. Similar relationships have also been found between anthocyanin concentration and antioxidant capacity. According to some researchers, the antioxidant capacity of a product is generally proportional to its TPC and anthocyanin content (Li et al., 2012; Tanguler et al., 2021). In this study, only KLB produced with black carrot juice included anthocyanin at a concentration of 391.31 mg/L (cyaniding‐3‐glucoside equivalent). Black carrot is characterized by an intense purple color and is known as a unique source of anthocyanin which has been shown to possess many health benefits (Pereira‐Caro et al., 2021). The main anthocyanins in black carrot are acylated derivatives (i.e., Cya‐3‐xylglc‐gal‐acylated‐sin, Cya‐3‐xylglc‐gal‐acylated‐fer, and Cya‐3‐xylglc‐gal‐acylated‐coum), but it also includes nonacylated (i.e., Cya‐3‐xyl‐glc‐gal and Cya‐3‐xyl‐gal) anthocyanin derivatives (Pereira‐Caro et al., 2021; Tanguler et al., 2021). Besides anthocyanins, black carrots also contain considerable amount of bioactive compounds such as phenolic acids, including chlorogenic, caffeic, sinapic, ferulic, and coumaric acids (Agcam et al., 2021; Algarra et al., 2014; Gu et al., 2019). It is worth noting that the sample containing black carrot juice had the highest values for all TFC, TMA, and AA characteristics. After evaluating all of the results, it is determined that the use of black carrot juice is appropriate for the manufacture of a nondairy probiotic functional water kefir‐like beverage enriched in vitamin, mineral, antioxidant, phenolic substance, and anthocyanin content. Although the apple used in the experiment had red skin, no anthocyanin was detected because the skin was completely removed from the fruit flesh prior to juice extraction. Moreover, anthocyanins were not found in the G‐KLB and GC‐KLB trials due to the use of white grape variety and the absence of anthocyanins in green cabbage. In a similar manner, the flesh of the 11 apple cultivars was determined to be absence of anthocyanins (Vieira et al., 2011) while flavonols and anthocyanins were not detected in green cabbages (Park et al., 2014). In the final product, the TFC and AA concentrations of apple‐based KLB were reported as 176.40 mg GA/L and 37.56 (%), whereas grape‐based KLB comprised 61.96 mg GA/L and 15.13 (%), respectively (Randazzo et al., 2016). The TFC and AA (by DPPH method) levels in black carrot extracts prepared with low‐ and high‐speed juicers ranged from 1750 to 1950 mg GA/L and 27.66 to 28.36 μmol Trolox/mL, respectively (Purkiewicz et al., 2020).

### 3.4 Color scores of water KLBs

WKG was originally defined as whitish to gray in color, however, this can be changed by the type of sucrose (table/refined or brown/unrefined sugar) and the color of various fruits and vegetables added to the culture medium (Cufaoglu & Erdinc, 2023; Guzel‐Seydim et al., 2021). Similarly, the color of water kefir or water kefir‐like beverages varies according to the same factors. Due to the use of refined white sugar in this study, the color of the beverages produced was mainly determined by the color of the fruits and vegetables used.

Significantly distinct CIELab color parameters were determined for water KLBs (*p* < .05, Table 1). In terms of color parameters, the black carrot‐based KLB had the lowest lightness (L*) value. BC‐KLB was characterized by having a high degree of darkness, almost black (L*: 1.03), due to its high anthocyanin content, which was responsible for its dark red and purple appearance. Furthermore, in all of the other trials, the lightness value was determined to be quite high, and the L* values were ranked from highest to lowest as GC‐KLB, G‐KLB, and A‐KLB, respectively. The findings revealed an inverse relationship between L* values and TFC values. This is due to the color of vegetables derived from flavonoid pigments including anthocyanidins, chalcones, and flavones (Park et al., 2014). Examining a* values reveals that a positive a* value was detected in all trials except for the GC‐KLB. Negative a* value indicates the presence of greenness in green cabbage‐based KLB, whereas redness is demonstrated by positive a* values in BC‐KLB, A‐KLB, and G‐KLB. The b* characteristic had positive (yellowness) values in all trials, but it is noteworthy that this value was fairly high in the drink made from apple juice when compared to other trials (*p* < .05). The strong yellow color in the A‐KLB is a result of the fact that the apple cultivar used in this study is Starking Delicious. This apple cultivar was referred to as yellow fleshed in other studies (Fang et al., 2022).

The *C*_ab_ and h° values were significantly differed among water KLBs (*p* < .05) and varied from 4.54 to 48.63 and 12.89 to 109.80, respectively. On the basis of these values, it can be concluded that GC‐KLB is light green, A‐KLB is yellow with medium intensity, BC‐KLB is dark red, and G‐KLB is yellow with low intensity. In a study involving the production of a beverage similar to water kefir from grape and apple juices, it was determined that the predominant color in both products was a pale yellow with low intensity (Randazzo et al., 2016).

### 3.5 Microbiological evaluation of juices and water KLBs

As shown in Table 2, all six microbial groups have been detected in all four unpasteurized FVJ samples. Particularly, GC and BC‐FVJ samples were found to have significantly higher levels (ranging between 3.07 and 6.05 log units) of all microbial populations than grape and apple juices. The high microbial counts of these juices can be attributed to their high pH levels (6.01 for GC and 6.32 for BC). Prior to pasteurization, black carrot (7.20 log cfu/mL) and green cabbage (6.04 log cfu/mL) juices presented significantly poorer hygiene conditions in terms of CB number than grape (1.47 log cfu/mL) and apple (2.90 log cfu/mL) juices. This could be because root (like a black carrot) and leafy (like a green cabbage) vegetables have the greatest risk of infection from manure application to soil (Davis & Kendall, 2012) and also the presence of soil in roots and leaves may increase the number of *Enterobacteriaceae* populations in samples by increasing nutrient availability and moisture retention (Ekman et al., 2021). All microbial groups were decreased to levels below detection limits (≤1 cfu/mL) after pasteurization treatment (data not shown).

In this investigation, the commercial WKG inoculant contained 10^7^ cfu/g of TMC and rod‐LAB, 10^6^ cfu/g of coccus‐LAB, 10^5^ cfu/g of TY, and 10^4^ cfu/g of non‐*Saccharomyces* yeasts (Table 2). In the study of Gökırmaklı and Güzel‐Seydim (2022), similar results for WKG were reported as 7.25 log cfu/g *Lactobacillus* spp., 5.82 log cfu/g *Lactococcus* spp., and 5.54 log cfu/g total yeasts.

Two hours after initiating fermentation with the activated WKG inoculant, a decrease of between 1.00 and 1.84 log cfu/mL was observed in all microbial groups compared to population levels recorded in the WKG inoculant. The use of various fruit and vegetable juices significantly altered the microbial populations of the kefir‐like beverages produced (*p* < .05, Table 2). It was discovered that the juices of grapes, apples, green cabbage, and black carrots were microbiologically complex and contained significant levels of TMC (6.06–7.77 log cfu/mL). At the end of the fermentation, the highest rod and coccus LAB numbers were determined in BC‐KLB as 8.04 log cfu/mL and 8.03 log cfu/mL, respectively, while the other KLBs hosted levels ranging from 5.22 log cfu/mL to 7.67 log cfu/mL for both LAB groups.

In all trials, there was an increasing trend in TY and non‐*Saccharomyces* numbers during fermentation. GC‐KLB contained 4.37 log cfu/mL of TY, whereas the other final beverages comprised between 6.79 and 7.79 log cfu/mL. Similarly, the level of non‐*Saccharomyces* yeasts at the end of fermentation was 4.03 log cfu/mL for GC‐KLB and varied between 6.25 and 7.43 log cfu/mL for the other fermented juices. *Enterobacteriaceae* was undetectable in all KLBs. These findings corroborate Guzel‐Seydim et al.'s (2021) and Cufaoglu and Erdinc (2023)'s assertion that LAB and yeasts are dominating the flora in water kefir. It is notable that the experiment relying on green cabbage juice showed the lowest level in all microbial groups. This result is not surprising since it is reported that different sulfur compounds (i.e., allyl isothiocyanate, methyl methanethiosulfinate, sinigrin, and dimethyl trisulfide) naturally present in cabbage have strong inhibition activity on the growth of primary yeasts and then bacteria (Arrais et al., 2022; Friedrich et al., 2022; Kyung & Fleming, 1997).

Similar results were obtained between microbial groups to those in this study when KLBs were produced from grape and apple juices. For apple and grape juices‐based KLBs, respectively, yeasts were 7.4 and 7.9 log cfu/mL, rod LAB were 7.7 and 7.9 log cfu/mL, coccus LAB were 7.4 and 8.0 log cfu/mL, and TMC levels were 7.5 and 7.9 log cfu/mL, while *Enterobacteriaceae* were below the detection limit (Randazzo et al., 2016).

### 3.6 Volatile organic compounds (VOCs) of W‐KLBs

A total of 96 different VOCs were detected by HS‐SPME‐GC–MS (Table 3). W‐KLBs were characterized by high aromatic complexities with compounds belonging to esters (28), terpenes and terpenoids (15), alcohols (12), aldehydes (10), sulfur compounds (10), carboxylic acids (9), ketones (5), alkanes (4), phenols (2), and other (1). Some VOCs are detected only in one experiment and not identified in others. In this regard, 17 compounds (13 terpenes and terpenoids, 1 ester, 1 aldehyde, 1 ketone, and 1 phenol) were detected only in the experiment BC‐KLB, whereas GC‐KLB contained 36 distinctive compounds (10 sulfur compounds, 9 alcohols, 7 aldehydes, 3 alkanes, 3 esters, 2 carboxylic acids, and 2 ketones). Similarly, eight compounds, seven of which were esters and one ketone, were detected solely in G‐KLB. In the kefir‐like beverage made with apple juice, five compounds (three esters, one carboxylic acid, and one terpene) not detected in other experiments were identified. The remaining 30 compounds were discovered in at least two of the W‐KLBs.

**TABLE 3:** Volatile compounds identified in water KLBs.

No.	Compound (μg/L)	A‐KLB	G‐KLB	BC‐KLB	GC‐KLB
Esters (28)					
1	Ethyl decanoate	2627.50	3038.50	45.46	21.89
2	Ethyl dodecanoate	1765.36	3069.46	24.05	nd
3	Ethyl octanoate	1472.90	nd	30.14	nd
4	Ethyl 9‐decenoate	727.11	2143.84	nd	nd
5	Isoamyl acetate	316.56	555.82	5.25	nd
6	Ethyl hexanoate	208.19	295.36	5.03	nd
7	Ethyl 9‐hexadecenoate	194.34	637.95	nd	nd
8	Phenethyl acetate	136.38	136.94	1.89	nd
9	Isoamyl octanoate	133.45	nd	nd	nd
10	Hexyl acetate	120.13	170.75	nd	1.80
11	Ethyl hexadecanoate	119.84	16.13	nd	nd
12	Ethyl tetradecanoate	101.01	157.05	2.11	nd
13	Ethyl linoleate	90.09	211.95	14.59	nd
14	Isoamyl decanoate	78.64	20.86	nd	nd
15	Ethyl Acetate	71.93	208.42	13.14	1.48
16	Ethyl nonanoate	32.20	nd	nd	nd
17	Isobutyl octanoate	20.03	nd	nd	nd
18	Ethyl octadecanoate	nd	1616.93	nd	nd
19	Ethyl E‐11‐hexadecenoate	nd	90.96	nd	nd
20	Ethyl 9α‐linolenate	nd	68.82	nd	nd
21	Decyl acetate	nd	42.31	nd	nd
22	Ethyl nonadecanoate	nd	33.71	nd	nd
23	Tetradecanol acetate	nd	19.84	nd	nd
24	Ethyl undecanoate	nd	18.66	nd	nd
25	Bornyl acetate	nd	nd	11.01	nd
26	Citronellyl formate	nd	nd	nd	5.46
27	[1.1’‐Bicyclopropyl]‐2‐octanoic acid. 2′‐hexyl‐. methyl ester	nd	nd	nd	2.53
28	trans‐2‐Hexenyl acetate	nd	nd	nd	1.82
	∑ Esters	8215.66	12554.26	152.67	34.98
Alcohols (12)					
29	Isoamyl alcohol	552.11	306.46	49.54	nd
30	Phenylethyl alcohol	397.79	105.98	31.75	nd
31	1‐Hexanol	47.69	23.05	nd	36.04
32	trans‐2‐Hexenol	nd	nd	nd	34.31
33	1‐Octen‐3‐ol	nd	nd	nd	15.43
34	(Z)‐2‐octen‐1‐ol	nd	nd	nd	8.00
35	1‐Nonanol	nd	nd	nd	7.15
36	3‐Cyclohexene‐1‐ethanol	nd	nd	nd	4.83
37	1‐Octanol	nd	nd	nd	4.44
38	2‐Hexadecanol	nd	nd	nd	2.62
39	2‐Methyl hexadecan‐1‐ol	nd	nd	nd	2.11
40	1‐Octyn‐3‐ol	nd	nd	nd	1.80
	∑ Alcohols	997.59	435.49	81.29	116.73
Carboxylic acids (9)					
41	Octanoic acid	88.58	30.95	30.16	9.77
42	Decanoic acid	82.44	39.04	17.74	8.14
43	7Z‐tetradecenoic acid	35.67	46.95	nd	nd
44	Lauric acid	22.34	43.48	8.39	4.81
45	3‐Decenoic acid	14.97	nd	nd	nd
46	Acetic acid	nd	nd	43.20	44.14
47	Pelargonic acid	nd	nd	2.55	3.04
48	Lactic acid	nd	nd	nd	3.23
49	Heptanoic acid	nd	nd	nd	1.61
	∑ Carboxlyic acids	244.00	160.42	102.04	74.74
Alkanes (4)					
50	Isobutane	53.44	21.72	4.13	1.60
51	3‐Phenylpropionitrile	nd	nd	nd	12.87
52	3‐Ethyl‐1.4‐hexadiene	nd	nd	nd	3.00
53	3‐Ethyl‐1.5‐octadiene	nd	nd	nd	1.71
	∑ Alkanes	53.44	21.72	4.13	19.18
Terpenes and terpenoids (15)					
54	α‐Farnesene	18.53	nd	nd	nd
55	(*E*)‐γ‐Bisabolene	nd	nd	108.29	nd
56	Geraniol	nd	nd	72.45	nd
57	Borneol	nd	nd	10.77	nd
58	β‐Caryophyllene	nd	nd	9.88	nd
59	*m*‐Cymenene	nd	nd	5.24	nd
60	3‐Gymnomitrene	nd	nd	4.65	nd
61	Linalool	nd	nd	4.51	nd
62	β‐Bisabolene	nd	nd	4.22	nd
63	β‐Farnesene	nd	nd	4.02	nd
64	α‐Terpineol	nd	nd	3.29	nd
65	Zingiberene	nd	nd	2.72	nd
66	*DL*‐Limonene	nd	nd	2.64	1.74
67	β‐Pinene	nd	nd	2.10	nd
68	cis‐Sesquisabinene hydrate	nd	nd	2.05	nd
	∑ Terpenes and terpenoids	18.53	nd	236.83	1.74
Aldehydes (10)					
69	Acetaldehyde	nd	19.26	1.76	nd
70	Nonanal	nd	nd	4.82	7.24
71	1‐Octanal	nd	nd	1.90	nd
72	2.4‐Decadienal	nd	nd	nd	1.39
73	(E.E)‐2.4‐heptadienal	nd	nd	nd	13.85
74	2‐Undecenal	nd	nd	nd	13.65
75	2‐Heptenal	nd	nd	nd	11.75
76	trans‐2‐Octenal	nd	nd	nd	8.80
77	2‐hexenal	nd	nd	nd	2.23
78	Hexanal	nd	nd	nd	2.09
	∑ Aldehydes	nd	19.26	8.48	61.00
Ketones (5)					
79	2‐Hydroxy‐cyclopentadecanone	nd	42.04	nd	nd
80	4‐Nonanone	nd	nd	1.78	1.84
81	β‐Damascenone	nd	nd	1.96	nd
82	Nerylacetone	nd	nd	nd	2.32
83	Sulcatone	nd	nd	nd	1.77
	∑ Ketones	nd	42.04	3.74	5.93
Phenols (2)					
84	2.4‐Di‐tert‐butylphenol	nd	17.97	2.96	nd
85	4‐Vinylguaiacol	nd	nd	4.29	nd
	∑ Phenols	nd	17.97	7.25	nd
Sulfur compounds (10)					
86	5‐(Methylsulfanyl) pentanenitrile	nd	nd	nd	40.57
87	Dimethyl trisulfide	nd	nd	nd	37.27
88	4.5‐Epithiovaleronitrile	nd	nd	nd	18.79
89	4‐(Methylsulfanyl)butanenitrile	nd	nd	nd	6.90
90	Allyl isothiocyanate	nd	nd	nd	5.45
91	Methyl disulfide	nd	nd	nd	4.55
92	Carbon disulfide	nd	nd	nd	3.01
93	Methanethiol	nd	nd	nd	2.66
94	β‐Phenethyl isothiocyanate	nd	nd	nd	2.40
95	Dimethyl pentasulfide	nd	nd	nd	1.95
	∑ Sulfur compounds	nd	nd	nd	123.55
Other (1)					
96	Biphenyl	23.22	nd	5.98	5.89
	∑ Other	23.22	nd	5.98	5.89

*Note*: Results indicate mean values of four measurements.

Abbreviations: A‐KLB, apple‐based KLB; BC‐KLB, black carrot‐based KLB; GC‐KLB, green cabbage‐based KLB; G‐KLB, grape‐based KLB; KLB, kefir‐like beverage; nd, not detected.

**FIGURE 1:**
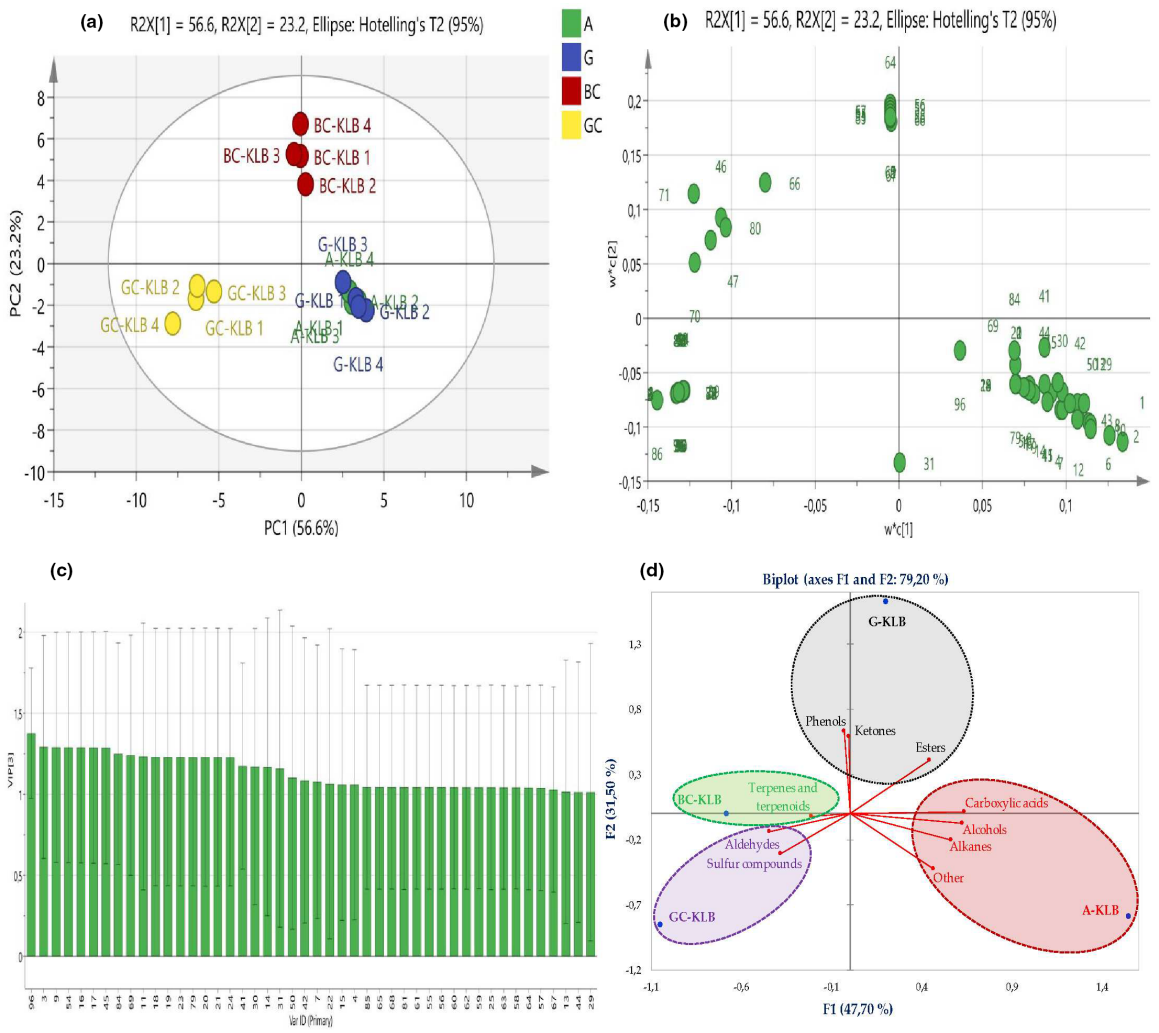
(a, b, and c) are the score scatter plot, loading scatter plot, and VIP score plot (with VIP > 1 of the volatile compounds) by PLS‐DA analysis, respectively. (d) is the PCA biplot graph of the total values of VOCs classified by chemical class. PLS‐DA, partial least squares‐discriminant analysis; VIP, variable importance in projection; VOC, volatile organic compound.

To know the contribution of the variables of the volatile compounds to the differentiation, the PLS‐DA model analysis was performed, where the goodness‐of‐fit parameter was R^2^X = 0.977, the model explanatory ability was R^2^Y = 0.998, and the predictive ability was *Q* ^2^ = 0.997. Figure 1 shows the multivariate analysis results for the four W‐KLBs in HS‐SPME‐GC–MS analysis, where the aroma compositional differences of the four KLBs could be seen more clearly. In Figure 1a, the cumulative contribution rate of PC1 and PC2 was 79.8% (PC1 56.60% and PC2 23.20%), indicating the PLS‐DA analysis was valid. Figure 1a showed the four kefir samples were distributed in four different positions by score scatter plot, suggesting the discrimination of the four W‐KLB's aroma compositions. By either PC1 or PC2, the A‐KLB and the G‐KLB were situated closer, and they were both situated far from the BC‐KLB and GC‐KLB. These suggested that regarding volatile composition, the grape‐KLB was more similar to apple‐KLB and they both were of greater differences from the black carrot‐ and green cabbage‐based kefir‐like beverages. This finding is consistent with the results of the sensory analysis, which revealed that panelists rated A‐KLB and G‐KLB as having very similar sensory properties. Nevertheless, based on either PC1 or PC2, the BC‐KLB was positioned as far away from the GC‐KLB as it was from the A‐KLB and G‐KLB, indicating that the black carrot‐based beverage differed in aroma from the green cabbage KLB as well as from the grape‐KLB and apple‐KLB.

Moreover, the PLS‐DA loading plot of Figure 1b revealed that bornyl acetate, (*E*)‐γ‐bisabolene, and geraniol were mainly related to the BC‐KLB. Acetic acid, 5‐(methylsulfanyl) pentanenitrile, and dimethyl trisulfide were mainly related to the GC‐KLB. Ethyl decanoate, ethyl dodecanoate, ethyl 9‐decenoate, ethyl octadecanoate, and 2‐hydroxy‐cyclopentadecanone were mainly associated with the G‐KLB. Ethyl decanoate, ethyl dodecanoate, isoamyl octanoate, octanoic acid, α‐farnesene, and isoamyl alcohol were mainly associated with the A‐KLB.

The variable importance in projection (VIP threshold >1) was used in important metabolites analysis in order to simplify data interpretation on the aroma profile of different W‐KLBs. Figure 1c showed a total of 46 compounds with VIPs greater than 1, including biphenyl, ethyl octanoate, isoamyl octanoate, α‐farnesene, ethyl nonanoate, isobutyl octanoate, 3‐decenoic acid, 2,4‐di‐tert‐butylphenol, acetaldehyde, ethyl hexadecanoate, 2‐hydroxy‐cyclopentadecanone, ethyl 9α‐linolenate, decyl acetate, etc. These compounds could be used as markers to discriminate aroma compositions of the four W‐KLBs. Regarding the PCA biplot (Figure 1d), two principal components explained 79.20% of the total variance, with the first component, F1, accounting for 47.70% and the second component, F2, accounting for 31.50%. As illustrated by Figure 1d, BC‐KLB was characterized by a higher concentration of terpenes and terpenoids, whereas A‐KLB was mainly explained by a higher concentration of alcohols, alkanes, carboxylic acids, and other metabolites. Similarly, the fragrance profile of G‐KLB was characterized by phenols, esters, and ketones, whereas the aroma profile of GC‐KLB was dominated by aldehydes and sulfur compounds. According to the predominating chemical classes, all kefir samples produced from various substrates were clearly distinguished from one another. Due to an increase in aldehydes and sulfur compounds, the GC‐KLB group was separated in the lower‐left quarter. Other groups were distinguished as follows: A‐KLB was located in the lower‐right quarter, G‐KLB in the upper‐right quarter, and BC‐KLB in the upper‐left quarter. These findings reveal that the fruits and vegetables used greatly affect the aroma and fragrance of W‐KLBs, and the sensory quality of the final beverage differs noticeably.

Esters were determined as the prevailing aroma group in both A‐KLB and G‐KLB. Moreover, terpenes and terpenoids were the chief aroma group in BC‐KLB, while sulfur compounds quantitatively represented the main aroma group in GC‐KLB samples. G‐KLB contained the highest amount of esters, followed by A‐KLB, BC‐KLB, and GC‐KLB, respectively (Table 3). On the other hand, the subtotal of alcohols was the highest in A‐KLB, followed by G‐KLB, GC‐KLB, and BC‐KLB, respectively. Remarkably, 13 of the 15 identified terpenes and terpenoids were found solely in BC‐KLB, while all of the identified sulfur compounds (10) were only detected in GC‐KLB. This observation is explained by the high sulfur content of *Brassica* vegetables (broccoli and cabbage), which has been reported to inhibit various yeast species, including *Saccharomyces cerevisiae* (Friedrich et al., 2022). The presence of sulfur compounds reduces the sensorial acceptability of the product, as evidenced by the sensory results of GC‐KLB. Terpenes, a large and diverse class of natural products that primarily consists of sesquiterpenes and monoterpenes, are the primary contributors to the organoleptic properties associated with numerous herbs, spices, citrus, and a majority of flowers and fruits (Wu et al., 2018). In accordance with this study's findings, Keskin et al. (2021) and Polat et al. (2022) identified terpenes as the most prevalent flavor group in fresh and dried black carrots. Terpenes were the most abundant ingredients in the lactic acid‐fermented black‐carrot beverage (Shalgam) after higher alcohols (Tanguler et al., 2017). In the present study, the main terpenes are (*E*)‐γ‐bisabolene, geraniol, borneol, β‐caryophyllene, *m*‐cymenene, linalool, *DL*‐limonene, β‐bisabolene, β‐farnesene, α‐terpineol, and β‐pinene. These results are consistent with the terpenes discovered in black carrots in some prior investigations (Keskin et al., 2021; Polat et al., 2022; Tanguler et al., 2017). Terpene volatiles, in general, serve an essential function in improving aromatic perception and positively affecting sensory impressions (Ferrão et al., 2022). This could be the main reason why the BC‐KLB obtained the highest score in terms of fragrance and aroma during sensory evaluation.

### 3.7 Sensory evaluation

**FIGURE 2:**
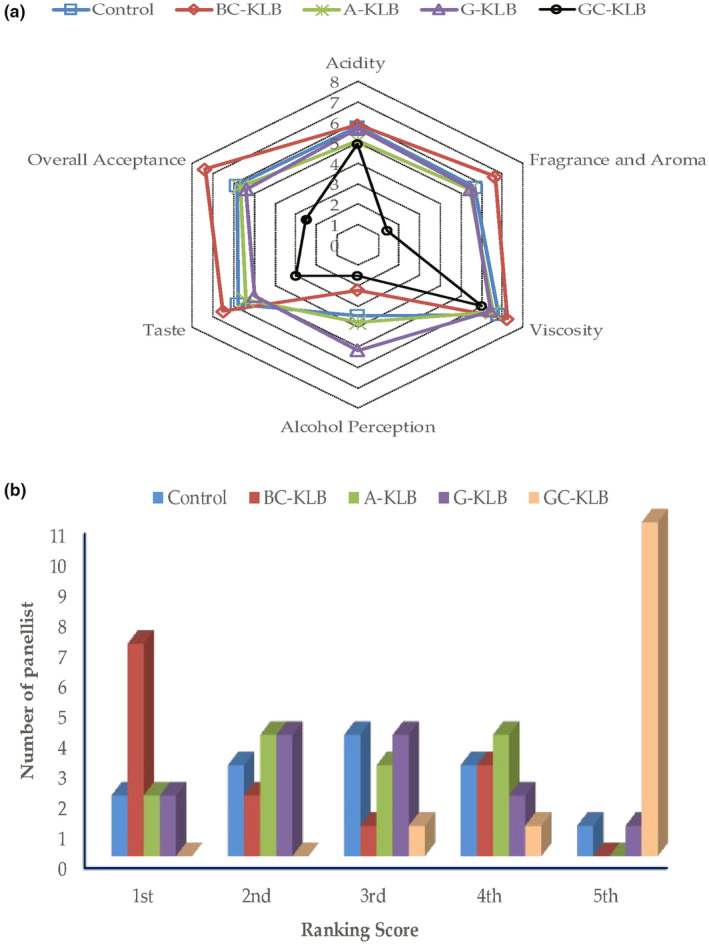
Illustration of sensory test results (a) as spider web graph and (b) preference test.

The purpose of the sensory evaluation of KLBs was to determine the sensory quality and potential differences between beverages made with four distinct juices. Furthermore, the sensorial quality of fruit–vegetable‐based KLBs was compared to that of a water kefir beverage (used as a control) produced with the same microbial mixture according to the manufacturer's instructions. The results from all descriptors showed that BC‐KLB received the highest scores for five (except alcohol perception) of the six characteristics evaluated by panelists (Figure 2a). In terms of taste, overall acceptance, fragrance, and aroma, the BC‐KLB treatment was markedly superior to the others, followed by the control group. Despite the fact that the A‐KLB received a higher score than the G‐KLB in terms of overall acceptability, the scores for both experiments were quite similar in all attributes, making it difficult to distinguish between them. On the other hand, the GC‐KLB beverage obtained the lowest score in all characteristics and had a poor flavor that could not be consumed. The raw form of green cabbage juice has definitely been proven to be unsuitable for the manufacturing of W‐KLBs. The main reason for this is that the sulfur compounds in cabbage juice adversely affect the fragrance and aroma of the product, resulting in a negative perception of the product right at the beginning, making it difficult to evaluate the other characteristics correctly. The use of various fruit and vegetable juices did not result in a substantial change in the viscosity values of the produced beverages, which were comparable to the control water kefir drink. Although BC‐KLB is the most appreciated product in terms of acidity, other experiments received comparable scores. The alcohol smell was highly perceived in G‐KLB, followed by A‐KLB, control, BC‐KLB, and GC‐KLB. Glucose is used faster than other sugars by yeasts; therefore, ethanol and CO_2_ levels are expected to be high in juices with high glucose content (Çevik et al., 2019; Laureys & De Vuyst, 2017). The alcohol perception characteristic was found to be consistent with the previously presented results (Table 2), indicating the initial sugar content of fresh juices and the alcohol content of the final product.

According to the results of both the scoring and preference tests, the KLB produced with black carrot juice was the one that consumers favored the most (Figure 2). Seven of 13 panelists placed BC‐KLB in the first rank, while 2 panelists placed it in the second position (Figure 2b). Equal numbers of panelists (two each) positioned the control, A‐KLB, and G‐KLB experiments in the first ranking. On the other hand, no one chose GC‐KLB as the first choice, but it was chosen as the last choice by 11 of the 13 panelists. Furthermore, control and G‐KLB were preferred once in the last rank. None of the judges selected BC‐KLB or A‐KLB as the worst beverages. As explained above, the A‐KLB and G‐KLB trials, which scored close in sensory analysis characteristics, showed a similar profile in the preference test and were ranked first and second by the same number of panelists. In conclusion, BC‐KLB was the most preferred beverage, and although A‐KLB was one step ahead of the remaining groups, there was no clear distinction among A‐KLB, G‐KLB, and the control group due to the closeness of their scores.

## 4 CONCLUSIONS

In this study, different fruit and vegetable juices were used as substrates in the fermentation carried out with commercial water kefir grains to meet the needs of the growing trend toward functional and healthy foods as well as various consumer typologies, involving vegans, vegetarians, and individuals with an intolerance or allergy to dairy products. Therefore, four different vegetable/fruit juices were used to produce novel, nondairy W‐KLBs with enhanced functional qualities. Because of the presence of sulfur compounds, it was determined that using green cabbage as a substrate was inappropriate for the manufacture of W‐KLB. On the other hand, the use of apple and grape juice demonstrates promising potential by resulting in comparable results to the control beverage. However, the findings of the investigation demonstrated that using black carrot in the process of producing water kefir‐like beverages yielded noticeably superior results, and it was concluded that this new, functional, nondairy beverage can meet the needs of consumers—vegans, vegetarians, and those who are allergic to or intolerant of dairy products in particular. Further research can explore the anthocyanin profile, compounds with antioxidant activity, characterization of lactic acid bacteria and yeast microbiota, probiotic characteristics, consumer acceptance, and commercial potential of the black carrot juice‐based product mentioned above.

## AUTHOR CONTRIBUTIONS

**Bilal Agirman:** Conceptualization (lead); data curation (equal); formal analysis (equal); investigation (equal); methodology (equal); software (lead); validation (equal); visualization (lead); writing – original draft (lead); writing – review and editing (lead). **Ilker Yildiz:** Formal analysis (equal); investigation (equal); methodology (equal). **Suleyman Polat:** Data curation (equal); formal analysis (equal); methodology (equal); validation (equal). **Huseyin Erten:** Conceptualization (supporting); funding acquisition (lead); project administration (lead); resources (equal); supervision (equal); writing – review and editing (supporting).

## CONFLICT OF INTEREST STATEMENT

The authors confirm that they have no conflict of interest to declare for this publication.

## Data Availability

The data that support the findings of this study are available on reasonable request from the corresponding author.
